# Suppression of Oxidative Stress and NFκB/MAPK Signaling by Lyophilized Black Raspberries for Esophageal Cancer Prevention in Rats

**DOI:** 10.3390/nu9040413

**Published:** 2017-04-22

**Authors:** Ni Shi, Fang Chen, Xiaoli Zhang, Steven K. Clinton, Xiaofei Tang, Zheng Sun, Tong Chen

**Affiliations:** 1 Division of Medical Oncology, Department of Internal Medicine, The Ohio State University, Columbus, OH 43210, USA; ni.shi@osumc.edu (N.S.); xiaoli.zhang@osumc.edu (X.Z.); steven.clinton@osumc.edu (S.K.C.); 2 The Arthur G. James Cancer Hospital and Richard J. Solove Research Institute, The Ohio State University, Columbus, OH 43210, USA; 3 College of Food Science and Nutritional Engineering, China Agricultural University, Beijing 100083, China; chenfangch@sina.com; 4 Center for Biostatistics, 1800 Cannon Drive, The Ohio State University, Columbus, OH 43210, USA; 5 Division of Oral Pathology, Beijing Institute of Dental Research, Stomatological Hospital & School of Stomatology, Capital Medical University, Beijing 100050, China; xftang10@ccmu.edu.cn (X.T.); sunzheng12@vip.sohu.com (Z.S.)

**Keywords:** black raspberries, esophageal cancer, oxidative stress, MAPK, NFκB

## Abstract

Research in the laboratory has shown that lyophilized black raspberries (BRB) significantly inhibit *N*-nitrosomethylbenzylamine (NMBA)-induced esophageal squamous cell carcinogenesis in rats. The objective of the present study is to characterize the underlying mechanism(s) of anti-cancer action of BRB in this preclinical animal model focusing on oxidative stress and its related oncogenic signaling pathways. Esophageal epithelial tissues were collected and assessed for markers of oxidative stress and nuclear factor κB (NFκB) and mitogen-activated protein kinase (MAPK). BRB reduced the incidence of esophageal cancer from 100% in NMBA-treated rats to 81.5% in rats treated with NMBA plus BRB (*p* < 0.05). Tumor multiplicity was reduced from 4.73 ± 0.45 tumors per esophagus in NMBA-treated rats to 1.44 ± 0.26 in rats treated with NMBA plus BRB (*p* < 0.001). The data indicated that NMBA treatment increased production of hydrogen peroxide and lipid hydroperoxide, reduced expression and activity of glutathione peroxidase and superoxide dismutase 2, and activated NFκB/MAPK signaling in rat esophagus. The study’s results show that BRB reverses oxidative stress and suppresses NFκB/MAPK pathways, which could be the mechanisms for esophageal cancer chemopreventive action of BRB in rats.

## 1. Introduction

Esophageal cancer is the sixth most common malignant neoplasm worldwide, with more than 90% of all cases being esophageal squamous cell carcinoma (ESCC) [1,2]. ESCC is strongly linked to exposure to chemical carcinogens, particularly associated with use of tobacco products [1,3]. The continued global expansion of tobacco consumption, coupled with high-risk dietary patterns, make ESCC a major public health threat for decades to come [1,4].

This study focuses upon the potential of chemopreventive strategies to inhibit the initiation and progression of ESCC and investigates their mechanisms of action using *N*-nitrosomethylbenzylamine (NMBA)-induced ESCC animal model [5,6,7,8]. In previous studies, it was found that inducible nitric oxide synthase (iNOS), cyclooxygenase-2 (COX-2), vascular endothelial growth factor (VEGF), c-Jun and human β-defensin 2 were associated with tumor development in animal model of esophageal carcinogenesis [6,7,9]. In parallel, we examined “food-based” prevention approach and found that lyophilized black raspberries (BRB) significantly inhibit NMBA-induced ESCC in rats.

Esophageal carcinogenesis is a multistage process characterized by morphological changes from normal epithelia to basal cell hyperplasia, dysplasia, carcinoma *in situ*, and ESCC. The anti-cancer function of BRB for ESCC includes regulations on inflammation, cell proliferation, angiogenesis, apoptosis, cell differentiation, and cell adhesion [6,7,10,11]. The anti-oxidative stress effects of berries have been studied under various conditions [12]. However, the association between oxidative stress and key oncogenic signaling contributed to esophageal carcinogenesis, and the effects of BRB on these molecular alterations have not been investigated. The present study focuses further upon elucidating the pleomorphic impacts of BRB on several additional pathways associated with esophageal carcinogenesis, which are particularly related to oxidant stress responses and related oncogenic signaling.

Oxidant stress is defined as an imbalance between byproducts of oxygen consumption and reactive metabolites, reactive oxygen species (ROS: ONOO^−^, O^2−^, hydrogen peroxide and lipid hydroperoxide), and their elimination antioxidants. This imbalance leads to accumulating damage to key macromolecules and underlies the initiation and subsequent progression of many cancers including ESCC [13,14]. Oxidative stress is linked with both chemical and environmental carcinogenesis [15,16]. In rodent models of ESCC, the elevated iNOS can lead to oxidative stress [16,17]. In 4-nitroquinoline 1-oxide (4-NQO)-induced esophageal tumor animal model, an increased level of ROS was observed [18]. DNA damage is the major outcomes from ROS [19]. The concentration of 8-hydroxy-2'-deoxyguanosine (8-OHdG), the most common modification of endogenous DNA base, in serum of patients with ESCC is significantly higher than healthy volunteers [20,21].

Glutathione peroxidase (GPx1, 2, 3, and 4) and superoxide dismutase 2 (SOD2) are critical enzymatic systems in production and decomposition of ROS [22,23]. ROS can activate downstream key transcription factors such as nuclear factor-erythroid 2-related factor-2 (Nrf2), nuclear factor κB (NFκB) and mitogen-activated protein kinase (MAPK) [24,25]. Alterations of NFκB and MAPK signaling are key oncogenic events during ESCC tumor promotion and progression [26,27].

In the present study, the hypothesis that BRB inhibits NMBA-induced ESCC in rats was tested through modulation of oxidative stress parallel to the suppression of related oncogenic signaling. The effects of BRB on GPx and SOD2 enzymatic systems as well as NFκB/MAPK activation were detected in NMBA-treated rat esophagus.

## 2. Materials and Methods

### 2.1. Chemicals and Reagents

NMBA was obtained from Ash Stevens (Detroit, MI, USA). Dimethyl sulfoxide (DMSO) was purchased from Sigma Chemical, Co. (St. Louis, MO, USA). The kits for glutathione, hydrogen peroxide (H_2_O_2_), and lipid hydroperoxide (LPO) assays were purchased from Cayman Chemical Company (Ann Arbor, MI, USA). The High-Capacity cDNA Reverse Transcription Kit and Fast SYBR Green Master Mix Kit were obtained from Applied Biosystems (Foster City, CA, USA). The antibodies of GPx1, p-extracellular signal–regulated kinases (ERK), p-stress-activated protein kinase/Jun amino-terminal kinases (SAPK/JNK), p-p38 MAPK, p-NFκB p65, p-nuclear factor of kappa light polypeptide gene enhancer in B-cells inhibitor, alpha (IκBα) and p-IκB kinase alpha/beta (IKKα/β) were purchased from Cell Signaling Technology (Danvers, MA, USA). Anti-8-oxoguanine (8-OxoG), GPx1, 2, 3, 4, and SOD2 antibodies were purchased from Abcam (Cambridge, MA, USA).

### 2.2. In Vivo Model of ESCC

Male F344 rats, four to five weeks old, were obtained from Harlan Sprague-Dawley (Indianapolis, IN, USA). The animals were housed three per cage under standard conditions and placed on AIN-76A synthetic diet (Dyets Inc., Bethlehem, PA, USA). Two weeks after initial housing, sixty rats were randomized into three experimental groups and treated immediately as follows. Rats in Group 1 were injected s.c. with 0.2 mL of a solution of 20% DMSO in water (the solvent for NMBA) three times per week for five weeks. Rats in Groups 2 and 3 were injected s.c. with 0.2 mL of NMBA (0.30 mg/kg body weight) in 20% DMSO: H_2_O three times per week for five weeks. Three days following the final NMBA treatment, rats in Group 2 were fed AIN-76A diet and rats in Group 3 were fed experimental diet (5% BRB) for the duration of the bioassay. The animals were fed an AIN-76A synthetic diet (powder) containing 20% casein, 0.3% d,l-methionine, 52% cornstarch, 13% dextrose, 5% cellulose, 5% corn oil, 3.5% AIN salt mixture, 1% AIN vitamin mixture, and 0.2% choline bitartrate (Dyets, Inc., Bethlehem, PA, USA). BRB were mixed into the diet (modified by reducing the concentration of cornstarch by 5% to maintain an isocaloric diet) for 25 min with a Hobart mixer (Troy, OH, USA). Fresh experimental and control diets were placed in glass feeding jars weekly and fed to the rats as described before [7]. At 35 weeks, all animals were euthanized by CO_2_ asphyxiation and subjected to gross necropsy. The esophageal tissues were collected as previously described [28]. This animal study protocol was reviewed and approved by the Institutional Animal Care and Use Committee of The Ohio State University (Protocol No.: 2009A0054-R2).

### 2.3. Immunohistochemistry

The formalin-fixed esophagus was cut into thirds and embedded in paraffin with the epithelium uppermost. Serial 4-µm sections were cut and mounted on superfrost plus slides (Histotechniques Laboratories, Powell, OH, USA). Tissues were subjected to immunohistochemistry staining using an 8-OxoG-specific antibody. The slides containing rat esophageal tissues were deparaffinized with histoclear and rehydrated in graded ethanol (100%–70%). Sections were incubated with 3% hydrogen peroxide, casein and goat serum, adivin and biotin, and then incubated with 8-OxoG antibody (Abcam, Cambridge, MA, USA) followed by goat anti-mouse biotinylated immunoglobin link, and streptavidin-horseradish peroxidase label. Finally, the sections were developed with diaminobenzidine. Reagents were supplied by BioGenex (Fremont, CA, USA). The 8-OxoG staining was evaluated by NIE-Elements BR 3.0 object count protocol (Nikon, Tokyo, Japan).

### 2.4. Real-Time RT-PCR

Total RNA was extracted from frozen esophagi using AllPrep DNA/RNA/Protein Mini Kit (Qiagen, Hilden, Germany) according to the manufacturer’s instructions. High capacity cDNA Reverse transcription Kit (Applied Biosystems, Foster City, CA, USA) was used to reverse-transcribe RNA into cDNA. Fast SYBR Green Master Mix (Applied Biosystems) was chosen for the amplification on a 7900HT Fast Real-Time PCR System (Applied Biosystems) and data were analyzed by Sequence Detection Systems Software version 2.3 (Applied Biosystems). PCR primers for GPx and SOD2 are listed in Table S1.

### 2.5. Western Blot Analysis

Proteins extracted from frozen esophageal epithelium were quantitated using a DC Protein Assay Kit (Bio-Rad Laboratories, Hercules, CA, USA) according to the manufacturer’s recommendations before separation. The Western blot was carried on XCellSureLock^®^ Mini-Cell and XCell II™ Blot Module (Invitrogen, Carlsbad, CA, USA). The immunoreactive bands were detected with an Immun-star^TM^ WesterC^Tm^ Kit (Bio-Rad Laboratories, Hercules, CA, USA) using Molecular Imager ChemiDoc XRS (Bio-Rad Laboratories).

### 2.6. Measurements of H_2_O_2_ and LPO

The concentrations of H_2_O_2_ and LPO in rat esophagus were measured using the Hydrogen Peroxide Kit and LPO Assay Kit (Cayman Chemistry, Ann Arbor, MI, USA), respectively, following the manufacturer’s instructions.

### 2.7. GPx and SOD2 Activity Assay

GPx activity was measured as described by Yan et al. [29]. Briefly, protein samples were incubated with 50 mM potassium phosphate buffer (pH 7.4) with 1 mM ethylenediaminetetraacetic acid (EDTA), 1 mM NaN_3_, 10 mM hydroperoxides (GSH), and 2.4 U/mL glutathione reductase (GR) at 25 °C for 5 min. The reduced form of nicotinamide adenine dinucleotide phosphate (NADPH) (1.5 mM) was added to the mixture and further incubated at 25 °C for 3 min. The reaction was then initiated by addition of 0.5 mM H_2_O_2_ or CuOOH. The activity was determined by the decrease in NADPH absorption at 340 nm for 6 min at 1 min intervals. The activity unit was defined as the amount of enzyme that caused the oxidation of 1 nmol of NADPH to NADP^+^ per minute at 25 °C. The specific activity was expressed as nmol/min/mg of total protein.

SOD activity was measured by the reduction of superoxide accumulation [30]. One unit of SOD activity was defined as the amount of enzyme that inhibits the rate of nitroblue tetrazolium reduction by 50%. SOD2 activity was calculated by subtracting Cu/Zn SOD activity from total SOD activity. The specific activity of SOD was expressed as U/mg of total protein.

### 2.8. GSSG/GSH and NADP^+^/NADPH Ratios

Glutathione disulfide (GSSG) and hydroperoxides (GSH) were measured using a Glutathione Assay Kit (Cayman Chemistry) according to the manufacturer’s instructions.

The nicotinamide adenine dinucleotide phosphate (NADP^+^)/NADPH (the reduced form of NADP^+^) measurement was described by Zhang et al. [31] with some modifications. Briefly, 10 μL extract was incubated with 200 μL of extraction buffer at 37 °C for 5 min and an absorbance measurement was taken at 340 nm. This reading measures the total amount of NADPH and NADH in the sample (A1). Another 10 μL extract was pre-incubated at 37 °C for 5 min in a reaction mixture containing 5.0 IU of glucose-6-phosphate dehydrogenase, 0.1 M Tris-HCl, pH 8.0, 0.01 M MgCl_2_, and 0.05% (*v*/*v*) Triton X-100. This reaction converted NADP^+^ to NADPH (A2). The reaction was initiated by the addition of glutathione disulfide (5 mM GSSG) to convert NADPH to NADP^+^. The absorbance of the mixtures at 340 nm was determined (A3). Subtraction of A3 from A1 represents the total amount of NADPH in the testing samples. The total amount of NADP^+^ was calculated by subtracting the A1 from A2.

### 2.9. Statistical Analysis

Tumor incidence (percentage of animals with tumors in each group) was analyzed using the χ^2^ test. The data of tumor multiplicity (number of tumors per animal), expression and activities of GPx and SOD2, GSSG/GSH and NADP^+^/NADPH ratios, H_2_O_2_ and LPO concentrations, and activations of MAPK and NFκB was analyzed and compared using two-way ANOVA Tukey’s multiple comparisons. All statistical analyses were carried out using GraphPad Prism 5.0 (GraphPad Software, San Diego, CA, USA). Differences were considered statistically significant at *p* < 0.05. All *p* values were two-sided.

## 3. Results

### 3.1. BRB Inhibits NMBA-Induced Tumor Development

As shown in Table 1, none of the DMSO-treated rats developed malignant lesions. BRB significantly reduced tumor incidence (*p* < 0.05) and tumor multiplicity (*p* < 0.001) in rats fed 5% BRB in Group 3 compared to those fed control diet in Group 2.

### 3.2. BRB Decreases Level of 8-OxoG

**Figure 1:**
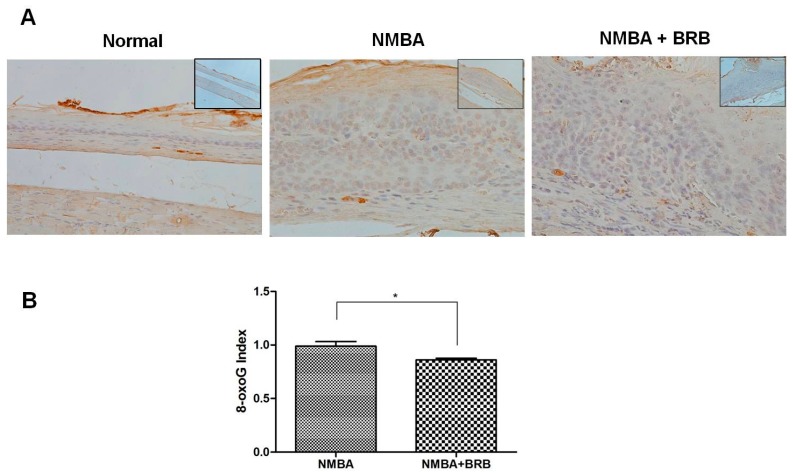
Black raspberries (BRB) decreases level of 8-OxoG. (**A**) Immunohistochemistry (IHC) staining of 8-OxoG in normal animals and rats treated with *N*-nitrosomethylbenzylamine (NMBA) or NMBA + BRB (200×). The upper right image is the version under 100×; and (**B**) statistical analysis of 8-OxoG staining. The values are expressed as mean; bars, ±SE. * *p* < 0.05.

As shown in Figure 1, antibody directed against 8-OxoG produced little staining in normal esophageal epithelium. The immunoreactivity of 8-OxoG was greater in NMBA-treated animals compared to normal animals. BRB significantly reduced the elevated level of 8-OxoG in esophageal epithelium (*p* < 0.05).

### 3.3. BRB Increases Expression Levels of GPx and SOD2

**Figure 2:**
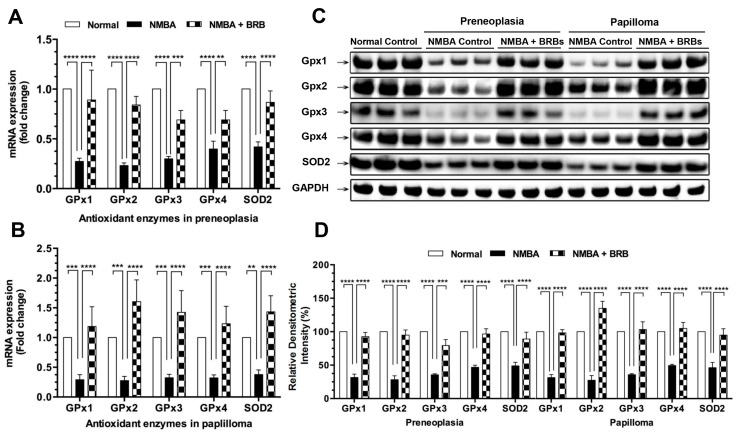
BRB increases expression levels of glutathione peroxidase (GPx) and superoxide dismutase 2 (SOD2). (**A**,**B**), mRNA expression of GPx and SOD2 in rat esophagus; (**C**,**D**), protein expression of GPx and SOD2 in rat esophagus. The values are expressed as mean; bars, ±SE. ** *p* < 0.01; *** *p* < 0.001; **** *p* < 0.0001.

The mRNA expression levels of GPx 1, 2, 3, 4, and SOD2 in esophageal preneoplasia following NMBA treatment were reduced by 27%, 23%, 30%, 40%, and 42%, respectively, when compared to normal animals (Figure 2A). The mRNA expression of GPx 1, 2, 3, 4, and SOD2 was significantly increased in rats fed BRB compared to those fed control diet. Moreover, BRB increased the mRNA expression of GPx 1, 2, 3, 4, and SOD2 in papilloma (Figure 2B). Western blot analysis also showed similar observations of GPx and SOD2 protein in both preneoplasia and papilloma (Figure 2C,D).

### 3.4. BRB Reduces H_2_O_2_ and LPO Concentrations

The elevated levels of H_2_O_2_ and LPO in esophageal preneoplastic and papillomatous lesions were observed in NMBA-treated animals fed control diet compared to normal animals (Table 2). BRB significantly decreased H_2_O_2_ concentrations in preneoplastic (78% reduction, *p* < 0.0001) and papillomatous lesions (61% reduction, *p* < 0.001). The elevated LPO concentration was reduced by 73% and 76% in esophageal preneoplasia and papilloma, respectively, in rats fed 5% BRB compared to those fed control diet.

### 3.5. BRB Enhances GPx and SOD2 Activities

Two metabolites, H_2_O_2_ and CuOOH, were employed in assays to define GPx enzyme activity. Based upon H_2_O_2_ concentration, NMBA treatment decreased GPx activity in esophageal preneoplasia (*p* < 0.001) and papilloma (*p* < 0.0001). In rats fed BRB, the GPx activity suppressed by NMBA was reversed in comparable tissues (*p* < 0.001). Similar results were observed when GPx activity was assessed using another substrate, CuOOH (Table 2). SOD2 activity was decreased by NMBA (*p* < 0.01) and increased by BRB (*p* < 0.05) in comparable tissues (Table 1).

### 3.6. BRB Reduces GSSG/GSH and NADP^+^/NADPH Ratios

The GSSG/GSH was increrased to 7.3- and 7.7-folds in preneoplasia and papilloma, respectively, following NMBA treatment when compared to normal animals (*p* < 0.001). In rats fed BRB, GSSG/GSH was decreased to 2.6- (*p* < 0.001) and 2.8-folds (*p* < 0.01) in preneoplasia and papilloma, respectively (Table 1). In analogous tissues, the NADP^+^/NADPH ratios were increrased to 8.7- and 11.3-folds in preneoplasia and papilloma, respectively, in rats treated with NMBA fed control diet (*p* < 0.0001). BRB significantly decreased NADP^+^/NADPH in comparable tissues (*p* < 0.001; Table 1).

### 3.7. BRB Suppressed Activation of NFκB and MAPK Signaling

**Figure 3:**
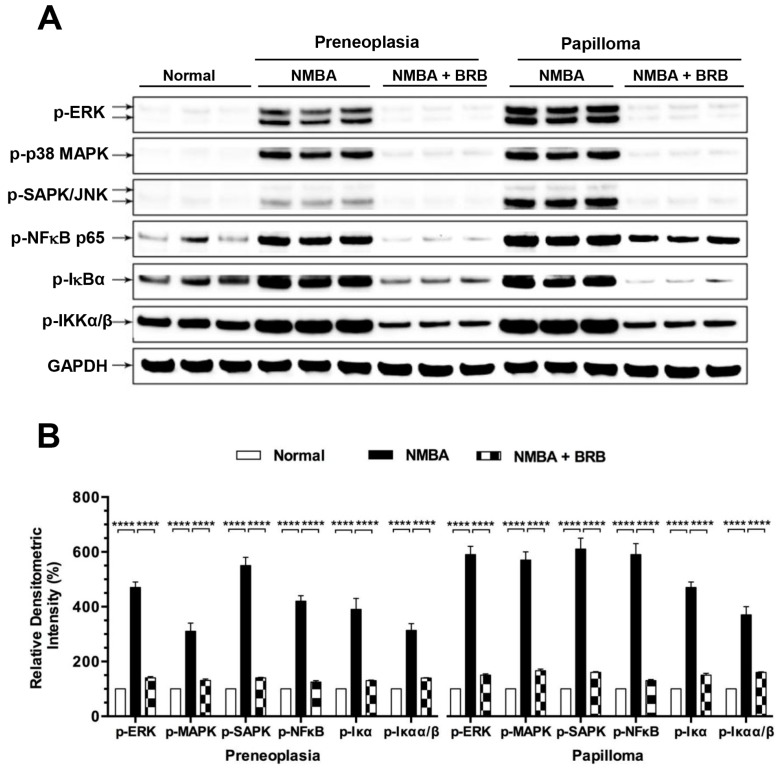
BRB suppressed activation of nuclear factor κB (NFκB) and mitogen-activated protein kinase (MAPK) signaling. (**A**) Western blot detected phosphorylation of extracellular signal–regulated kinases (ERK), p38 MAPK, stress-activated protein kinase/Jun amino-terminal kinases (SAPK/JNK), NFκB-p65, nuclear factor of kappa light polypeptide gene enhancer in B-cells inhibitor, alpha (IκBα), and IκB kinase alpha/beta (IKK)α/β in rat esophagus; and (**B**) quantitative analysis of western blot. The values are expressed as mean; bars, ±SE. **** *p* < 0.0001.

**Figure 4:**
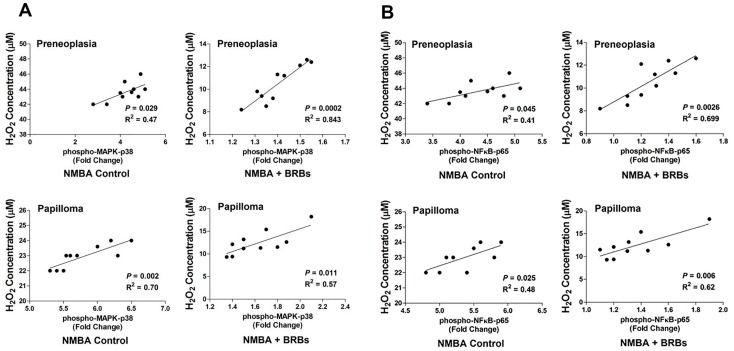
Correlations between H_2_O_2_ production and phosphorylation of NFκB p65 (**A**) or p38 MAPK (**B**) in NMBA-treated rat esophagus.

NMBA treatment increased phosphorylation of NFκB-p65, IκBα, IKKα/β, p38 MPAK, ERK and SAPK/JNK in esophageal preneoplasia to 4.2-, 3.9-, 3.1-, 3.1-, 4.7-, and 5.5-folds compared to normal animals (Figure 3). BRB significantly suppressed the phosphorylation of the above signaling towards the levels which were observed in normal animals (*p* < 0.0001). Similar effects of BRB on NFκB and MAPK signaling were detected in papilloma. Moreover, we found that phosphorylation of NFκB (Figure 4A) and MAPK (Figure 4B) was positively correlated with H_2_O_2_ concentration in rat esophagus.

## 4. Discussion

Esophageal cancer induced by chemical and physical agents involves multi-step processes. Oxidative damage resulting from ROS generation could participate in all stages of the ESCC process. This study observed that NMBA exposure suppressed expression and activity of GPx and SOD2, increased concentrations of H_2_O_2_ and LPO, GSSG/GSH, and NADP^+^/NADPH, and activated NFκB/MAPK signaling in rat esophagus (Table 3). BRB inhibited NMBA-induced esophageal tumorigenesis through reverse oxidative stress in parallel to suppression of NFκB/MAPK activations.

Oxidative stress occurs as a response to increased ROS or decreased antioxidants and leads to a failure to prevent ROS-induced oxidative damage. H_2_O_2_ and LPO are two important indicators for ROS. H_2_O_2_ is conversed from superoxide anion (O_2_^−^) [32]. LPO is oxidized by ROS from polyunsaturated fatty acids [33]. In rats treated with NMBA, H_2_O_2_ and LPO concentrations were increased in both esophageal preneoplasia and papilloma. GPx and SOD2 play critical roles in production and decomposition of H_2_O_2_ and LPO. SOD2 catalyzes the conversion of superoxide anion (O_2_^−^) to molecular oxygen and H_2_O_2_. H_2_O_2_ is subsequently removed by GPx through oxidation of two molecules of GSH to produce H_2_O and GSSG, which can be reduced by glutathione reductase with consumption of NADPH [34,35]. In accordant with these unbalanced ROS caused by NMBA treatment in esophageal tissues, expression levels of GPx and SOD2 were significantly decreased compared to normal animals. To examine whether the alterations in expression of GPx and SOD2 are consistent with their enzyme functions, we assessed the activities of GPx and SOD2, GSH, NADPH and their oxidized species, GSSG, and NADP^+^ in rat esophagi. GSH, a tripeptide containing a free thiol group of cysteine, is a major tissue antioxidant that donates reducing equivalents (H^+^ + e^−^) for GPx-catalyzed reduction of H_2_O_2_ and LPO [36]. By providing an electron, GSH itself becomes reactive and reacts with another GSH to form GSSG, an oxidized state of glutathione. When cells are exposed to increased oxidative stress, GSSG will accumulate and the ratio of GSSG to GSH will increase. NADPH plays a key role in retaining GSH in cells [37]. Increased ROS can result in a decrease in GSH and NADPH, and converts these into oxidized GSSG and NADP^+^, respectively. The GSSG/GSH and NADP^+^/NADPH ratios, therefore, serve as indicator of oxidative stress to assess the effects of antioxidant intervention strategies [38]. Our results show that NMBA induce oxidative stress during esophageal carcinogenesis in rats.

Mammalian defenses against oxidant stress include dietary components including nutrients (vitamin C, vitamin E, and selenium) and various phytochemicals with the ability to chemically quench ROS and other free radicals [12]. BRB is an abundant source of flavonoid compounds (ellagic acid, ferulic acid, coumaric acid, quercetin, and anthocyanins), vitamins (vitamins A, C, and E, and folic acid), minerals (calcium, potassium, selenium, and zinc), and phytosterols (β-sitosterol, campesterol, and stigmasterol) [39]. Numerous studies show that BRB exhibits chemopreventive effects in oral, esophageal, colon, skin and breast cancers in preclinical models and/or in humans [6,7,9,40,41,42]. Multiple bioactive phytochemicals in nature food products may have additive or synergistic activity with greater overall safety for cancer prevention. The mechanisms by which BRB inhibit tumorigenesis in the rat esophagus include reducing inflammation, cell proliferation, and angiogenesis, stimulating apoptosis, cell differentiation, and cell adhesion [10].

In previous studies, 20 components in BRB classified to four categories were identified and quantified: anthocyanins, ellagitannin, ellagic acid and derivatives, and flavonols [40]. Anthocyanins are the major phenolic components in BRB by dry weight (84.2%). Cyaniding-3-rutinosides (C3R) is the main anthocyanin in BRB (58.2%), followed by cyaniding-3-xylorutinoside (18.2%), cyanidin-3-glucoside (C3G; 4.9%), cyaniding-3-sambubioside (C3S; 2.1%), and pelargonidin-3-rutinoside (0.8%). The non-anthocyanin phenolics include ellagitannins (11.5%), ellagic acid and derivatives (0.7%), and flavonols (3.6%). Anthocyanins have been shown to exhibit antioxidant activities in vivo and in vitro [43,44]. C3G can inhibit oxidative stress in preclinical skin cancer model and stimulate biosynthesis of GSH in mice with hepatic oxidative damage [45,46]. Our studies indicated that C3R, C3G, and C3S inhibit cell proliferation and oncogenic signaling pathways in ESCC cell lines [40]. The dietary BRB has been shown to reduce urinary 8-OHdG in patients with Barrett’s Esophagus [5]. The mechanism of BRB on antioxidant defense system, however, has not been fully elucidated. In the current study, it was found that BRB enhanced GPx and SOD2, reduced ROS including H_2_O_2_, LPO, and decreased GSSG/GSH and NADP^+^/NADPH, indicators of oxidative stress. These results demonstrate that BRB inhibit rat esophageal tumor development through reducing oxidative stress.

**Figure 5:**
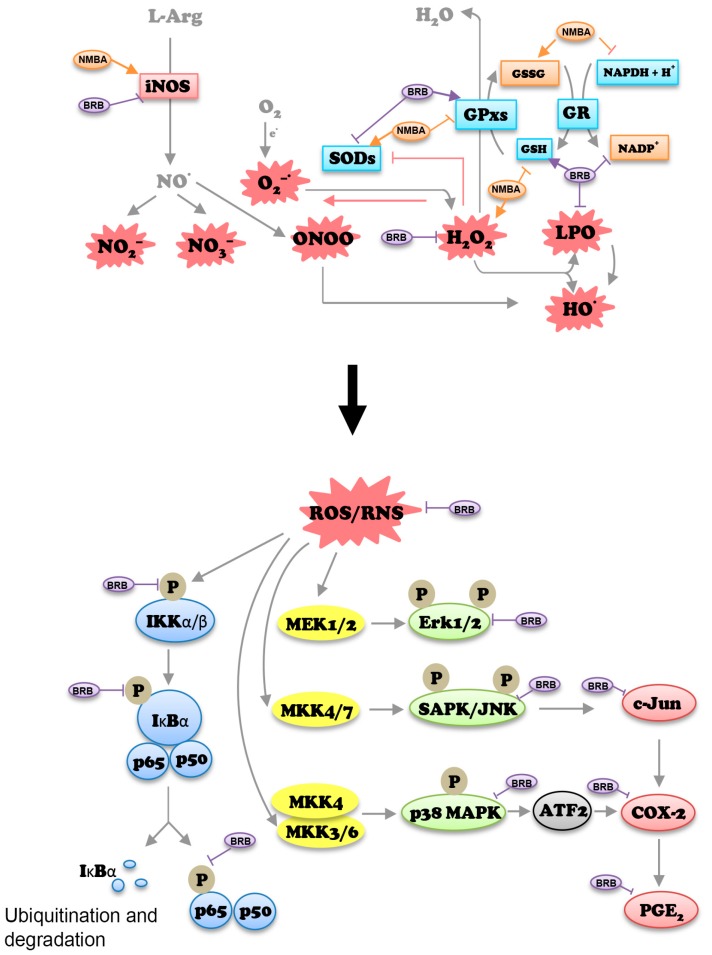
Possible mechanisms of action of BRB in esophageal cancer prevention. Upper figure, BRB modulates oxidative stress/antioxidant pathway; lower figure, BRB inhibits MAPK and NFκB activation. Arrows indicate activation and T-lines indicate inhibition. L-Arg: L-arginine; iNOS: inducible nitric oxide synthase; ONOO: Peroxynitrite; GSSG: glutathione disulfide; NADPH: nicotinamide adenine dinucleotide phosphate reduced form; GSH: hydroperoxides using glutathione; GR: glutathione reductase; ROS/RNS: reactive oxygen species and reactive nitrogen species; P: phosphorylation; MEK: mitogen-activated protein kinase kinase; MKK: mitogen-activated protein kinase kinase; Erk: extracellular-signal-regulated kinases; COX-2: cyclooxygenase 2; PGE_2_: prostaglandin E2.

ROS acts as second messenger molecule to transduce signaling cascades that control diverse cellular events such as proliferation, apoptosis and inflammation [24]. ROS can activate MAPK family members including p38 MAPK and JNK, which subsequently activate genes involved in cellular proliferation [47]. NFκB is a pleiotropic transcription factor modulating inflammatory responses, innate and adaptive immunity, cell proliferation, differentiation, and survival [48]. Activation of NFκB occurs in response to a wide spectrum of extracellular stimuli. Activation of NADPH oxidases induced by enhanced ROS production can lead to NFκB activation [48,49]. In esophageal cancer, H_2_O_2_ produced by Barrett’s, inflammatory and other parenchymal cells can activate MAPK and NFκB that increase cell proliferation and DNA damage, thereby contributing to esophageal tumorigenesis [50]. H_2_O_2_ can also induce the activation of NFκB, IκBα, and p38 MAPK in epithelial cells [51]. In this study, it was found that NMBA-induced elevated H_2_O_2_ in esophageal tissues was significantly reduced by BRB, and activation of NFκB/MAPK was suppressed in NMBA-treated animals fed BRB compared to those fed control diet. Moreover, a positive correlation between H_2_O_2_ and NFκB-p65/p38 MAPK in both esophageal preneoplasia and papilloma was ovserved. These data suggested that chemical carcinogen-induced oxidative stress may activate NFκB and MAPK signaling pathway during esophageal carcinogenesis. BRB has potent esophageal cancer prevention potential through parallel impact on both oxidative stress/antioxidant system and NFκB/MAPK pathways (Figure 5).

The authors of the present study conducted extensive research in chemoprevention of ESCC with BRB [6,7,9,40]. In these previous studies, it was found that BRB significantly suppresses COX-2, iNOS, human β-defensin 2 (HBD-2) and NFκB in NMBA-treated animals. The data also indicate that the proinflammatory mediators interleukin-1β (IL-1β), IL-6, and tumor necrosis factor-α (TNF-α) are upregulated by NMBA. NFκB acts as a central mediator under inflammatory condition through its transcriptional regulation on cytokines, chemokines and adhesion molecules [52,53]. TNF-α, COX-2, and iNOS are under stream targets of NFκB. Moreover, TNF-α, iNOS, and IL-1β can be regulated by another important transcriptional factor, nuclear factor erythroid 2-related factor 2 (Nrf2) [54]. In this study, we also found that the mRNA expression of Nrf2 encoding gene *NFE2L2* was elevated from 1.52 fold at week 6 (*p* < 0.05) to 2.10 fold at week 29 (*p* < 0.001) in rats treated with NMBA compared to normal animals (data not shown). The overexpression of Nrf2 is associated with metastasis and unfavorable outcomes in patients with esophageal cancer [55]. Numerous studies show that there are functional cross-talks between Nrf2 and NFκB in oxidative stress and inflammation related carcinogenesis, e.g., the inhibition of Nrf2 can exacerbate NFκB activity; on the other hand, NFκB can modulate transcription and activity of Nrf2 [54]. Inflammation derived oxidative stress is one of the important mechanisms in inflammation associated cancers. The current study assessed oxidative stress related molecular events during esophageal carcinogenesis. It is demonstrated that the NMBA-induced oxidative stress is reversed by BRB. In a mouse model of azoxymethane/dextran sodium sulfate-induced colorectal cancer, deficiencies in antioxidant genes such as GPx3 increase the number of tumors in mice [56]. The findings from the current study combined with our previous observations demonstrate that BRB inhibits tumor development in rat esophagus, at least in part, through the reversal of oxidative stress and suppression of NFκB/MAPK activation, in parallel to the inhibition of inflammation. Further studies are needed to elucidate inflammation and oxidative stress in esophageal carcinogenesis focusing on effects of BRB on Nrf2 and interplays between Nrf2 and NFκB.

## 5. Conclusions

In summary, this study demonstrates for the first time that oxidative stress is induced in NMBA-treated rat esophagus. Moreover, this study showed that the level of H_2_O_2_ is correlated with NFκB p65 and p38 MAPK in the esophagus of rats treated with NMBA and dietary BRB. Although the precise mechanisms remain to be elucidated, the modulation of oxidative stress by BRB appears to be associated with changes in NFκB/MAPK activation. This study suggests that BRB may offer an advantage in cancer prevention by targeting multiple signaling pathways.

## Figures and Tables

**Table 1:** Effect of lyophilized black raspberries (BRB) on *N*-nitrosomethylbenzylamine (NMBA)-induced tumorigenesis in the rat esophagus.

Group	NMBA ^a^	Diet	No. of Rats	Tumor Incidence (%)	Tumor Multiplicity Mean ± SE ^b^	Tumor Volume (mm^3^) ^c^ Mean ± SE
1	−	AIN-76A	20	0	0	0
2	+	AIN-76A	20	100	4.73 ± 0.45	7.91 ± 2.67
3	+	5% BRB	20	81.5 ^d^	1.44 ± 0.26 ^e^	9.12 ± 3.76

^a^ “−“, without NMBA treatment and “+”, with NMBA treatment; ^b^ SE, standard error; ^c^ Tumor volume calculated as length × width × depth × π/6 assuming a prolate spheroid shape; ^d^ Significantly lower than Group 2 as determined by χ^2^ test (*p* < 0.05); ^e^ Significantly lower than Group 2 as determined by analysis of variance (*p* < 0.001).

**Table 2:** Effects of BRB on oxidative stress.

	Normal	Preneoplasia		Papilloma	
		NMBA ^h^	NMBA + BRB ^i^	NMBA ^h^	NMBA + BRB ^i^
H_2_O_2_^a^	7.2 ± 0.9	44.1 ± 3.8 ****	9.6 ± 1.2 ****	23.7 ± 2.3 ****	10.2 ± 1.1 ***
LPO ^b^	10.5 ± 2.5	74.0 ± 7.3 ****	20.1 ± 5.7 ****	97.1 ± 4.1 ****	22.8 ± 4.8 ****
GPx (H_2_O_2_) ^c^	85.4 ± 3.5	19.4 ± 4.6 ****	72.6 ± 8.0 ****	29.8 ± 2.7 ****	85.9 ± 10.4 ****
GPx (CuOOH) ^d^	213.4 ± 33.5	55.4 ± 17.8 ****	205.9 ± 13.1 ****	79.9 ± 16.6 ****	231.5 ± 16.4 ****
SOD2 ^e^	0.39 ± 0.0	0.17 ± 0.0 ***	0.31 ± 0.0 **	0.18 ± 0.0 ***	0.33 ± 0.0 **
GSSG/GSH ^f^	1.0 ± 0.0	7.3 ± 1.0 ****	2.6 ± 0.8 ****	7.7 ± 1.4 ****	2.8 ± 0.6 ****
NADP^+^/NADPH ^g^	1.0 ± 0.0	8.7 ± 0.8 ****	2.0 ± 0.3 ****	11.3 ± 1.1 ****	2.8 ± 0.3 ****

^a^ H_2_O_2_ concentration is expressed as μM/mg; ^b^ lipid hydroperoxide (LPO) concentration is expressed as μM/mg; ^c^ glutathione peroxidase (GPx) activity (H_2_O_2_ as substrate) is expressed as nmol/min/mg; ^d^ GPx activity (CuOOH as substrate) is expressed as nmol/min/mg; ^e^ superoxide dismutase 2 (SOD2) activity is expressed as U/mg; ^f^ the concentration ratio of glutathione disulfide (GSSG) to hydroperoxides (GSH); ^g^ the concentration ratio of nicotinamide adenine dinucleotide phosphate (NADP^+)^ to the reduced form of NADP^+^ (NADPH); ^h^ Significantly different compared to normal animals (*** *p* < 0.001, **** *p* < 0.0001); ^i^ Significantly different compared to NMBA-treated animals fed control diet (** *p* < 0.01, *** *p* < 0.001, **** *p* < 0.0001).

**Table 3:** Effects of BRB on oxidative stress and NFκB/MAPK in NMBA-induced rat esophageal carcinogenesis.

	NMBA	BRB
Tissue oxidative stress index		
8-OxoG IHC staining	↑	↓
Anti-oxidative enzymes		
GPx	↓	↑
SOD2	↓	↑
Cell oxidative stress index		
H_2_O_2_ and LPO	↑	↓
GSSG/GSH	↑	↓
NADP^+^/NADPH	↑	↓
NFκB pathway		
p65, IκBα, IKKα/β	↑	↓
MAPK pathway		
p38, ERK and SAPK/JNK	↑	↓

IHC, immunohistochemistry; NFκB, nuclear factor κB; IκBα, nuclear factor of kappa light polypeptide gene enhancer in B-cells inhibitor, alpha; IKK α/β, IκB kinase alpha/beta; MAPK, mitogen-activated protein kinase; ERK, extracellular signal–regulated kinases; SAPK/JNK, stress-activated protein kinase/Jun amino-terminal kinases; “↑”, increase; “↓”, decrease.

## Supplementary Materials

The following are available online at www.mdpi.com/2072-6643/9/4/413/s1, Table S1: Nucleotide sequences of the primers used to assay gene expression by Real-time PCR.

## Abbreviations

ESCC, esophageal squamous cell carcinoma; BRB, black raspberries; NMBA, *N*-nitrosomethylbenzylamine; MAPK, mitogen-activated protein kinase; NFκB, nuclear factor κB; SOD2, superoxide dismutase 2; GPx, Glutathione peroxidase; H_2_O_2_, hydrogen peroxide; LPO, lipid hydroperoxide; 8-OxoG, 8-oxoguanine; ROS, reactive oxygen species; GSH, hydroperoxides using glutathione; GSSG, glutathione disulfide; NADP^+^, nicotinamide adenine dinucleotide phosphate; NADPH, the reduced form of nicotinamide adenine dinucleotide phosphate; JNK, Jun amino-terminal kinases; SAPK, stress-activated protein kinases; iNOS, inducible nitric oxide synthase; COX-2, cyclooxygenase-2; VEGF, vascular endothelial growth factor; IκBα, nuclear factor of kappa light polypeptide gene enhancer in B-cells inhibitor, alpha; IKKs, IκB kinases; ERK, extracellular-signal-regulated kinases; EDTA, ethylenediaminetetraacetic acid; GR, glutathione reductase; GAPDH, glyceraldehyde-3-phosphate dehydrogenase; C3R, cyaniding-3-rutinosides; C3G, cyanidin-3-glucoside; C3S, cyaniding-3-sambubioside; IL-1β, interleukin-1β; TNF-α, tumor necrosis factor-α; HBD-2, human β-defensin 2; 8-OHdG, 8-hydroxy-2’-deoxyguanosine; Nrf2, nuclear factor erythroid 2-related factor 2.
