# Supplementary material for: Suppression of Oxidative Stress and NFκB/MAPK Signaling by Lyophilized Black Raspberries for Esophageal Cancer Prevention in Rats
Source: Nutrients. 2017 Apr 22;9(4):413. doi: 10.3390/nu9040413 (PMC5409752; doi:10.3390/nu9040413)
Supplement: Supplementary file 1 [file nutrients-09-00413-s001.pdf]

**Supplemental Table S1**. Nucleotide sequences of the primers used to assay gene expression by real-time PCR.

| Gene | Forward | Reverse |
|---|---|---|
| GPx 1 | 5′-ACGATGTTGCCTGGAACTTT-3′ | 5′-TCGATGTCAATGGTCTGGAA-3′ |
| GPx 2 | 5′-TAAGTGGGCTCAGGCCTCTCT-3′ | 5′-GGTCATAGAAGGACTTGGCAATG-3′ |
| GPx 3 | 5′-ACAGGAAGAGCTTGCACCAT-3′ | 5′-CTCCTGGTTCCTGTTTTCCA-3′ |
| GPx 4 | 5′-CAGTGAGGCAAGACCGAAGT-3′ | 5′-CTGCTTCCCGAAGTGGTTAC-3′ |
| SOD2 | 5′-ACGTTGGATGCTGTGCTTTCTCGTCTTCAG-3′ | 5′-ACGTTGGATGTTCTGCCTGGAGCCCAGATAC-3′ |
